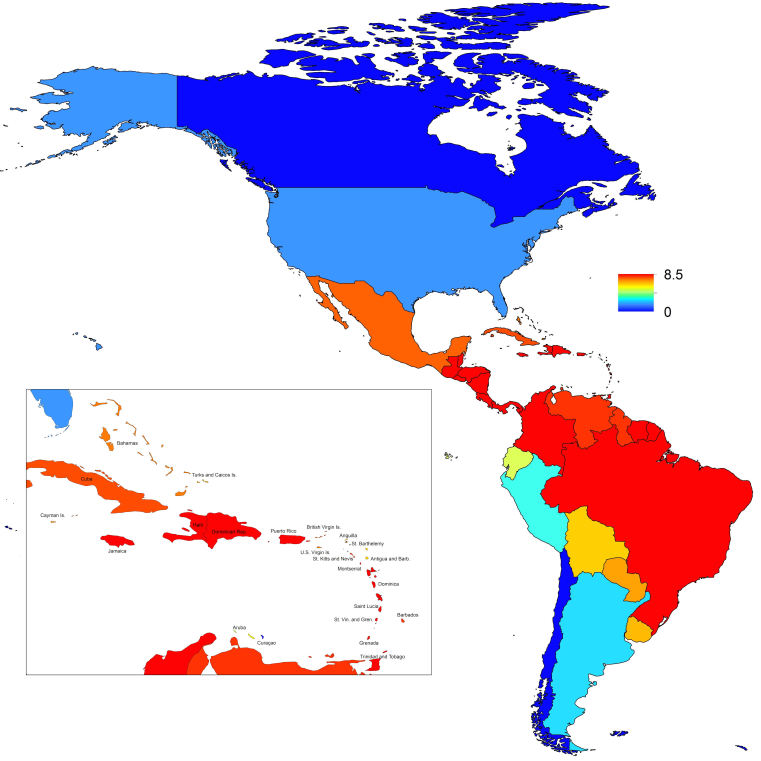# Estimating Drivers of Autochthonous Transmission of Chikungunya Virus in its Invasion of the Americas

**DOI:** 10.1371/currents.outbreaks.a4c7b6ac10e0420b1788c9767946d1fc

**Published:** 2015-02-10

**Authors:** T. Alex Perkins, C. Jessica E. Metcalf, Bryan T. Grenfell, Andrew J. Tatem

**Affiliations:** Department of Biological Sciences and Eck Institute for Global Health, University of Notre Dame, Notre Dame, Indiana, USA; Fogarty International Center, National Institutes of Health, Bethesda, Maryland, USA; Department of Ecology and Evolutionary Biology, Princeton University, Princeton, New Jersey, USA; Fogarty International Center, National Institutes of Health, Bethesda, Maryland, USA; Department of Ecology and Evolutionary Biology, Princeton University, Princeton, New Jersey, USA; Department of Geography and Environment, University of Southampton, Southampton, UK; Flowminder Foundation, Stockholm, Sweden

**Keywords:** Aedes, arbovirus, Chikungunya, epidemic, invasion, mathematical model, mosquito, R0, seasonality, TSIR

## Abstract

Background Chikungunya is an emerging arbovirus that has caused explosive outbreaks in Africa and Asia for decades and invaded the Americas just over a year ago. During this ongoing invasion, it has spread to 45 countries where it has been transmitted autochthonously, infecting nearly 1.3 million people in total. Methods Here, we made use of weekly, country-level case reports to infer relationships between transmission and two putative climatic drivers: temperature and precipitation averaged across each country on a monthly basis. To do so, we used a TSIR model that enabled us to infer a parametric relationship between climatic drivers and transmission potential, and we applied a new method for incorporating a probabilistic description of the serial interval distribution into the TSIR framework. Results We found significant relationships between transmission and linear and quadratic terms for temperature and precipitation and a linear term for log incidence during the previous pathogen generation. The lattermost suggests that case numbers three to four weeks ago are largely predictive of current case numbers. This effect is quite nonlinear at the country level, however, due to an estimated mixing parameter of 0.74. Relationships between transmission and the climatic variables that we estimated were biologically plausible and in line with expectations. Conclusions Our analysis suggests that autochthonous transmission of Chikungunya in the Americas can be correlated successfully with putative climatic drivers, even at the coarse scale of countries and using long-term average climate data. Overall, this provides a preliminary suggestion that successfully forecasting the future trajectory of a Chikungunya outbreak and the receptivity of virgin areas may be possible. Our results also provide tentative estimates of timeframes and areas of greatest risk, and our extension of the TSIR model provides a novel tool for modeling vector-borne disease transmission.

## Introduction

Chikungunya is a painful affliction characterized by fever, arthralgia, and varying other symptoms 1 ^,^ 2. It is caused by Chikungunya viruses (CHIKV), which are vectored between people primarily by either *Aedes aegypti* or *Ae. albopictus* mosquitoes 3, depending on local vector ecology 1 and viral strain 4. Outbreaks of Chikungunya have been highly explosive in a variety of contexts, ranging from tropical islands 5 to temperate mainlands 6. A large portion of cases are thought to be symptomatic 2, making these outbreaks highly conspicuous, readily documentable, and of serious concern to public health.

After its discovery in the 1950s, CHIKV was recognized as the etiological agent in outbreaks that occurred throughout Africa, India, and Southeast Asia over the next several decades 1 ^,^ 7 ^,^ 8. The last ten years, however, have seen an alarming number of outbreaks globally, increased importation to new areas, autochthonous transmission in Europe, and most recently invasion and establishment in the Americas 8 ^,^ 9. The first known autochthonous cases of CHIKV in the Americas were reported on December 5, 2013, and occurred on the island of Saint Martin in the Caribbean 10. Its spread has since continued throughout the Caribbean and into mainland South and North America 9. The sequence of invasion from one country in the Americas to another has received considerable attention from modelers and appears to be somewhat predictable based on flight information, distance between countries, and climatic suitability 11 ^,^ 12 ^,^ 13 ^,^ 14.

There have also been attempts to model the dynamics of the early stages of establishment within a country, yielding estimates of probabilities of autochthonous transmission upon introduction 12 ^,^ 15 and the basic reproductive number *R*_0_ 13, which is defined as the expected number of secondary infections caused by a single primary infection in a susceptible population. Given the importance of the mosquito vector in transmitting CHIKV, it is to be expected that the potential for autochthonous transmission should depend greatly on local climatic and ecological conditions 16 ^,^ 17 and that this potential should therefore vary greatly in time and space. Efforts to quantify transmission potential to date have relied on empirically derived descriptions of how different components of vectorial capacity depend on weather-related covariates such as temperature and precipitation 12 ^,^ 15, yet there has been very little confirmation that these relationships are predictive of realized patterns of transmission. There has also been scant consideration of susceptible depletion and its feedback on to transmission dynamics via herd immunity, which should be important given the strong protective immunity that Chikungunya infection confers 2 ^,^ 3 and the high seroprevalence observed following outbreaks 5 ^,^ 18 ^,^ 19.

To fill these gaps among models that have been applied to the CHIKV epidemic in the Americas thus far, we adapt the time-series susceptible-infectious-recovered (TSIR) framework 20 for modeling CHIKV transmission dynamics. Originally developed for measles, the TSIR framework has been applied to a variety of infectious diseases since 21 ^,^ 22 ^,^ 23 ^,^ 24 ^,^ 25 ^,^ 26 ^,^ 27 and offers a convenient way to model and estimate susceptible build up and depletion and spatial and temporal variation in transmission. We describe our application of this model to weekly case reports from countries in the Americas during the first year of CHIKV invasion there. In doing so, we establish direct relationships between climatic drivers and transmission, and we propose a platform for future work that will allow for inference of more nuanced links between transmission and putative drivers and for forecasting the continued spread of CHIKV throughout the Americas.

## Methods

The goal of our analysis was to understand drivers of spatial and temporal variation in the potential for autochthonous transmission, rather than drivers of pathogen movement and case importation. Consequently, we used a model that accounts for the transmission process locally but that ignores the process of pathogen movement between countries. The question of CHIKV dispersion and importation in the Americas has been addressed previously 11 ^,^ 12 ^,^ 13 ^,^ 14 and is something that could be incorporated into our framework in the future.

Model

Our model pertains to weekly incidence, which is denoted *I_i,t_* for week *t* in country *i*. We denote the number of residents of *i* that are susceptible during week *t* as *S_i,t_*. Given that the duration of infectiousness is expected to be about five days on average 12, the remainder of the population is assumed to have recovered and gained immunity within a week, so *R_i,t_* = *N_i_* - *S_i,t_* - *I_i,t_*. In doing so, we assume that the total population size *N_i_* is static and that births and deaths are negligible on the timeframe over which the model is applied. The duration of the incubation period of the virus in humans is expected to be between three and seven days 2, so we assume that cases in week *t* derive from susceptible people in week *t*-1. Due to the presence of a vector, the period of time separating successive cases, or the serial interval, is relatively prolonged and variable. To account for this, we introduce a modification to the standard TSIR framework that allows for an arbitrary specification of the serial interval distribution.

To account for this distributed time lag between successive cases, we treated the effective number of infectious people during the time interval in which transmission occurs as

\begin{equation*}I_{i,t}' = \sum_{n=1}^5 \omega_n \left( I_{i,t-n} + \iota_{i,t-n} \right) \qquad , \qquad \qquad \qquad \qquad \qquad \qquad \qquad \qquad \qquad \qquad \qquad (1)\end{equation*}

where *I_i,t-n_* are cases acquired locally and *ι_i,t-n_* are imported cases. The coefficients that weight contributions of infectious people *n* weeks ago to infections in the current week are calculated according to

\begin{equation*}\omega_n = \frac{1}{7} \int_{7(n-1)}^{7n} F(\tau+7)-F(\tau) \, d\tau \qquad , \qquad \qquad \qquad \qquad \qquad \qquad \qquad \qquad \qquad \qquad \qquad (2)\end{equation*}

where *F* is the distribution function of the serial interval and *τ* is a dummy variable. This formulation assumes that the timing of cases within a week is uniform and that a case on day *t* arose from a case on day *t*-*τ* with probability *f*(*τ*), where *f* is the density function corresponding to *F*. We chose a functional form and parameters for *f* and *F* consistent with a previously published serial interval distribution for CHIKV 13. Assuming a gamma distribution and applying the method of moments to the mean and standard deviation reported in 13, we used values of the shape and rate parameters for the serial interval distribution of 14.69 and 0.64, respectively. Applying these numbers to eqn. (2) using the integrate function in R 28 and normalizing resulted in values of ω_1,...,5_ = 0.011, 0.187, 0.432, 0.287, and 0.083. A schematic depiction of the calculation of *I'*_*i,t*_ based on *I_i,t-n_* and ω*_n_* is shown in Fig. 1.

**Schematic representation of the calculation of effective numbers of infectious people, I'i,t.**
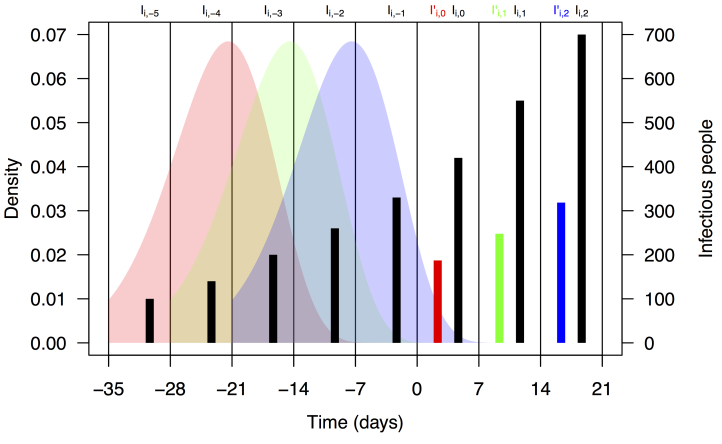
Black bars represent observed weekly case numbers, and red, green, and blue bars in weeks 0-2 represent effective numbers of infectious people in three consecutive weeks. Colored shapes show the serial interval distributions used in the calculation of ω*_n_* and then in the calculation of *I'*_*i,t*_ in each of weeks 0-2. Weekly case numbers were chosen for pedagogical purposes and do not reflect empirical data.

Consistent with a frequency-dependent formulation of the TSIR model for directly transmitted pathogens 23, we modeled the dynamics of the infectious class as

\begin{equation*}I_{i,t} = \beta_{i,t} \frac{ {I_{i,t}'} ^ \alpha}{N_i} S_{i,t-1} \qquad , \qquad \qquad \qquad \qquad \qquad \qquad \qquad \qquad \qquad \qquad \qquad (3)\end{equation*}

where *β*_*i,t*_ is a transmission coefficient for country *i* in week *t*. Under this formulation, *β*_*i,t*_ is related to the basic reproductive number, *R*_0_, in country *i* in week *t* by

\begin{equation*}R_0(i,t) = \sum_{n=1}^5 \omega_n \beta_{i,t+n} \qquad . \qquad \qquad \qquad \qquad \qquad \qquad \qquad \qquad \qquad \qquad \qquad (4)\end{equation*}

The transmission coefficient *β_i,t_* is assumed to implicitly account for a number of factors, including the probabilities of transmission from infectious people to susceptible mosquitoes and from infectious mosquitoes to susceptible people, the ratio of mosquitoes to people, mosquito longevity beyond the pathogen's incubation period in the mosquito, and the rate at which adult female mosquitoes feed on blood 27. Another assumption of this formulation is that encounters between mosquitoes and people are well mixed, which while potentially problematic for modeling mosquito-borne pathogen transmission 29, can be accounted for phenomenologically by inclusion of the mixing parameter α in [0,1] 30 ^,^ 31. Dynamics of the susceptible and recovered classes follow from eqn. (3), the assumption of recovery within one week, and the assumption of a static population, yielding *S_i,t_* = *S_i,t-1_* - *I_i,t_* and *R_i,t_* = *R_i,t-1_* + *I_i,t-1_*.

Data

The centerpiece of our analysis were weekly numbers of Chikungunya cases on a national scale for countries in the Americas. At the time that we conducted our analysis, there were 1,293,836 cases reported over 61 weeks in 50 countries. Of these, 1,185,728 were suspected cases, each of which corresponded to an individual who sought medical treatment and was diagnosed with Chikungunya based on their presentation of symptoms. An additional 101,651 cases were confirmed by either PCR, serology, or laboratory culture. The remaining 6,457 cases were deemed imported based on travel histories. We obtained data for the first ten weeks from Project Tycho 32, which in turn obtained them from the Agence Régionale de Santé, and for the remaining 51 weeks from the Pan American Health Organization's website (www.paho.org).

In addition to case numbers, we utilized data on monthly temperature and precipitation averaged at a national scale from 1 km × 1 km gridded data. These data were obtained from WorldClim (www.worldclim.org), and represent interpolated meteorological station data on temperature and precipitation from the 1950-2000 period, processed to create climatological monthly averages that represent "typical" conditions 33. To obtain weekly temperature and precipitation values, we assigned monthly values to weeks that fell entirely within a month and took a weighted average in the event that a week spanned two months. We obtained country-level population estimates from the Central Intelligence Agency World Factbook (www.cia.gov/library/publications/the-world-factbook/). At the onset of CHIKV invasion, we assumed that the entire population of each country was susceptible, with the number of susceptibles in each country decreasing each week thereafter by the numbers of suspected and confirmed cases.

Estimating drivers of transmission

Given data on weekly cases and a generative model for those data, we estimated the mixing parameter α and relationships between local transmission coefficients *β*_*i,t*_ and two putative drivers of transmission: temperature and precipitation. To do so, we rearranged terms in eqn. (3) to arrive at the regression equation

\begin{equation*}\ln(I_{i,t}) - \ln (S_{i,t-1}) + \ln (N_i) = \ln(\beta_{i,t}) + \alpha \ln (I_{i,t}') \qquad , \qquad \qquad \qquad \qquad \qquad \qquad \qquad \qquad \qquad \qquad \qquad (5)\end{equation*}

where

\begin{equation*}\ln(\beta_{i,t}) = f(T'_{i,t},P'_{i,t}) + \epsilon \qquad , \qquad \qquad \qquad \qquad \qquad \qquad \qquad \qquad \qquad \qquad (6)\end{equation*}

*T'_i,t_* and *P'*_*i,t*_ are moving averages of temperature and precipitation in country *i* in weeks *t*-5 through *t*-1, and ε is a normally distributed random variable with mean zero. Regarding the functional form of *f*(*T'*_*i,t*_,*P'*_*i,t*_), we assumed a quadratic relationship,

\begin{equation*}f(T'_{i,t},P'_{i,t}) = a_0 + a_1 T'_{i,t} + a_2 P'_{i,t} + a_3 {T'_{i,t}}^2 + a_4 {P'_{i,t}}^2 \qquad , \qquad \qquad \qquad \qquad \qquad \qquad \qquad \qquad \qquad \qquad \qquad (7)\end{equation*}

because of the general expectation in the literature of a unimodal, and often quadratic, relationship between climatological variables and various aspects of vectorial capacity 12 ^,^ 16 ^,^ 17 ^,^ 47. To select among subsets of this model with the possibility of some coefficients equal to zero, we used the stepAIC function in the MASS package 35 in R 28. This applied both forward and backward selection to yield a model minimizing the Akaike Information Criterion and estimates of best-fit values of its coefficients. Because weeks in which either *I_i,t_* or *I'_i,t_* equalled zero were not informative in the regression, we performed this analysis only for country-weeks in which these conditions were not violated. We furthermore excluded weeks for which *I'_i,t_* < 1 to preserve a single case as a lower bound for generating autochthonous transmission. We considered *I_i,t_* to include both suspected and confirmed cases and *ι_i,t_* to represent imported cases. To examine patterns of variation in transmission predicted by the best-fit model, we computed values of *β_i,t_* based on the fitted model for 53 countries in the Americas in each of 52 weeks in a year with a typical temperature and precipitation regime.

**Table 1. Significance tests of terms in the regression (eqns. (5)-(7)) of log incidence (ln(Ii,t)) on temperature (T'i,t), precipitation (P'i,t), and the log of a weighted average of incidence in the previous five weeks (ln(I'i,t)).** 

Term	Parameter	Estimate	Standard error	*t*	*p*
Intercept	*a*_0_	-25.66	6.702	-3.829	1.46 × 10^-4^
*T'_i,t_*	*a*_1_	2.121	0.5733	3.699	2.41 × 10^-4^
*P'_i,t_*	*a*_2_	1.188 × 10^-2^	4.190 × 10^-3^	2.836	4.76 × 10^-3^
*T'_i,t_* ^2^	*a*_3_	-4.231 × 10^-2^	1.198 × 10^-2^	-3.533	4.51 × 10^-4^
*P'_i,t_* ^2^	*a*_4_	-2.882 × 10^-5^	1.116 × 10^-5^	-2.582	1.01 × 10^-2^
ln(*I'_i,t_*)	α	0.7413	3.285 × 10^-2^	22.571	< 2 × 10^-16^

**Partial residual plots of the fitted regression for temperature (top left), precipitation (top right), ln(I'i,t) (bottom left), and the intercept (bottom right).**
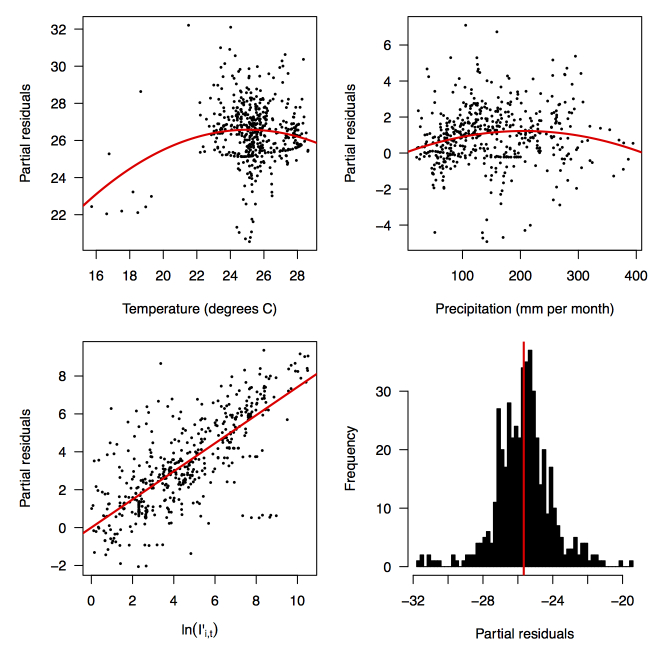

**Relationship between predicted and observed cases in 484 country-weeks on a log-log scale.**
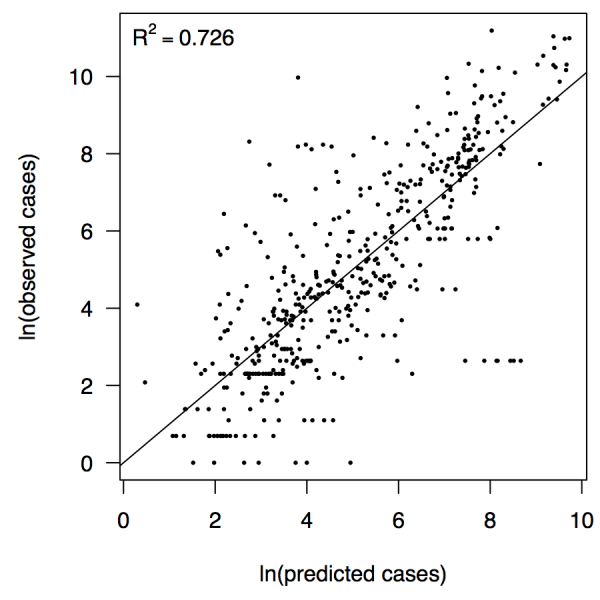
The line shows a one-to-one relationship for context.

## Results

Performing a regression of incidence against temperature and precipitation according to eqns. (5)-(7) yielded significant associations between transmission and linear and quadratic terms for temperature and precipitation (*F*_5,478 _= 256.9, *p *< 2.2 × 10^-16^) (Table 1). There was likewise strong support for a mixing parameter less than one, with a best-fit estimate of α = 0.74 (*t* = 22.57, *p *< 2 × 10^-16^). Although models with fewer terms were fitted and compared, the full model in eqn. (7) had the lowest AIC value and was thus supported as the best model by that criterion. Partial residual plots provided an indication of the extent to which each variable accounted for different portions of overall residual variation (Fig. 2). Overall, the model accounted for 72.6% of variation in incidence among country-weeks, as determined by adjusted *R* ^2^ (Fig. 3).

**Fitted relationship for f(T'i,t,P'i,t), which models the influence of weekly mean temperature and precipitation on the transmission coefficient βi,t.**
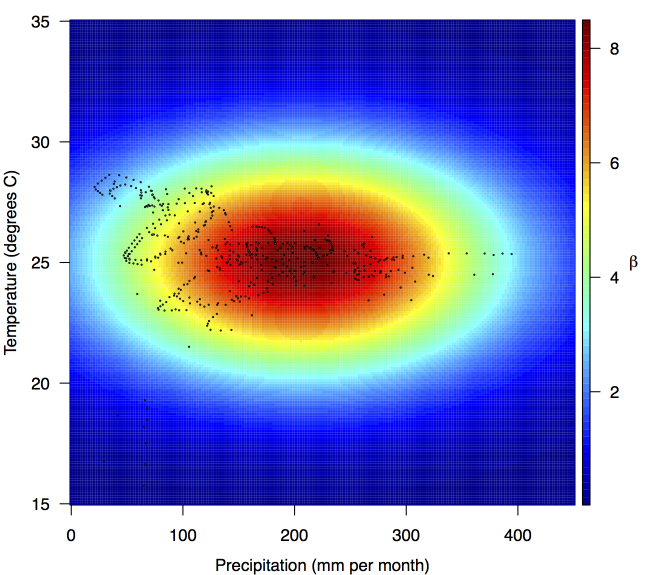
Points show temperature and precipitation values associated with country-weeks with positive incidence that were used in the regression.

Projections of the fitted model indicated that the transmission coefficient, and *R*_0_, should be highest at a temperature of 25 °C and monthly precipitation of 206 mm (Fig. 4). Most country-weeks that experienced autochthonous transmission of CHIKV fell within approximately 3 °C of the temperature optimum but across a large swath of monthly precipitation values (Figs. 2 & 4). Applying the best-fit model to temperature and precipitation data from all 52 weeks in 53 countries showed that the timing and duration of high-transmission seasons are projected to vary substantially across countries (Fig. 5). Such differences mimic clear latitudinal patterns in the seasonality of temperature and, in some areas, precipitation. In general, countries at high and mid latitudes were projected to have the highest potential for Chikungunya transmission from April through November and countries at low latitude from November through April, although there were of course some exceptions to these general patterns (Fig. 5). In addition to geographic variation in seasonality, the best-fit model also projected that mid-latitude countries should generally have higher transmission potential than those at latitudinal extremes (Fig. 6-9). Some outliers included countries with substantial areas of high-altitude terrain, such as Ecuador and Peru.

**Seasonal patterns of projected weekly R0 by country.**
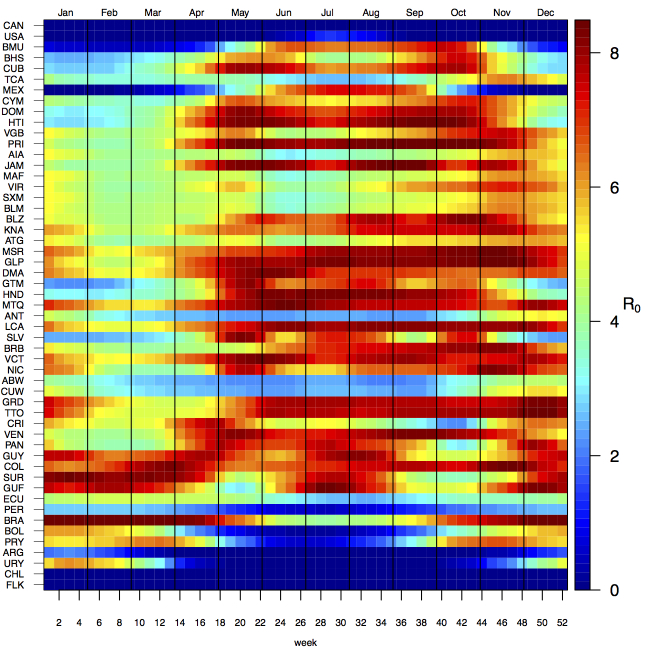
Countries are sorted by the latitudes of their capital cities.

**Variation in the range of projected weekly values of R0 by country.**
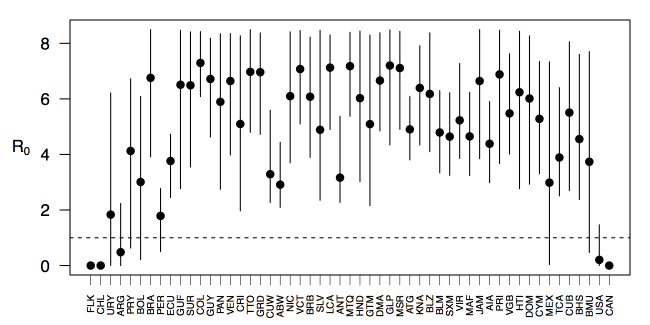
Points show mean values across the year and line segments span the ranges of weekly values. Countries are sorted by the latitudes of their capital cities.

## Discussion

The ongoing epidemic of Chikungunya throughout the Americas is nearing 1.3 million cases and is showing no signs of abating. In many ways, the present time is a critical juncture in the pathogen's invasion and in the public's response to it. Because CHIKV has been spreading in the Americas for over a year, there are sufficient data to begin analyzing its spread and learning about drivers thereof, as we have demonstrated in the present analysis. At the same time, there are many more millions of people at risk, so improving the capacity to forecast, prepare for, and mitigate outbreaks is paramount. In the present study, we have made several advances towards this goal.

Building on successful application of TSIR models to childhood and other diseases 20 ^,^ 21 ^,^ 22 ^,^ 23 ^,^ 24 ^,^ 25 ^,^ 26 ^,^ 27, we have proposed this framework as a potentially useful tool for modeling CHIKV transmission. Application of this method to CHIKV is reasonable based on a number of similarities between these pathogens, including the development of strong protective immunity, a reasonably short period of infectiousness, and the potential to rapidly infect (and induce immunity in) large numbers of people. At the same time, application of this method to CHIKV requires some important considerations. First, incorporation of frequency-dependent transmission and dependence on climatic drivers is critical 22 ^,^ 24. Second, the serial interval for vector-borne diseases is necessarily much longer than it is for directly transmitted diseases due to incubation of the pathogen in the vector and the possibility of prolonged transmission over multiple feeding cycles of the vector. By proposing a formulation of the TSIR model similar to an autoregressive moving average time series model, we have provided a new way to accommodate this important feature of vector-borne disease biology without unnecessarily aggregating data temporally and thus potentially compromising information content of the data.

A powerful feature of the TSIR framework is that it reveals variation in transmission and provides a clear and uncomplicated way of statistically associating that variation with putative drivers of transmission. Our analysis of 484 country-weeks of data indicated that there were significant relationships between country-level transmission of CHIKV and typical temperature and precipitation regimes. The concordance of these inferred relationships with previous knowledge is encouraging, because these relationships were apparent in our analysis only because of their demonstrated relationship with variation in transmission and not because of a priori assumptions. The inferred association between temperature and transmission is reasonable due to its height in the 20-30 °C range, although the inferred optimum of 25 °C is lower than some studies would suggest 12 ^,^ 16 but consistent with others 17. The relationship between precipitation and transmission that we inferred is also biologically plausible, as extremely low precipitation would make for insufficient mosquito breeding habitats, and too much could flood eggs from breeding habitats or make people less likely to store water and thereby reduce habitat for the aquatic stages of the *Aedes aegypti* mosquito that has been implicated in the current outbreak. For both of these relationships, it is worth bearing in mind that values of the covariate climate data that we used reflect national and long-term averages, and values in more localized areas where transmission occurs will vary considerably and exhibit inter-annual variations. Consequently, our estimates of optimal conditions for transmission are not directly comparable to estimates derived from local studies. Nonetheless, the relationships that we inferred are biologically plausible and, in the spirit of forecasting, predictive of variation in transmission.

Applying inferred relationships between transmission and putative drivers thereof to comprehensive spatial and temporal data on those drivers offers a means to anticipate future hotspots of transmission in space and time. On the one hand, such predictions could provide local public health agencies with an estimate of timeframes over which they may be more likely to experience outbreaks due to elevated autochthonous transmission, allowing time to mobilize resources for increased vector control or hospital beds 36. On the other hand, considering these predictions in a regional context could provide insight about when and from where imported cases are likely to appear. Combining this information with knowledge of when the potential for autochthonous transmission should be highest would be most valuable 37. Patterns of coupling in the timing of heightened transmission between different areas also have implications for regional persistence 38 ^,^ 39. Provided that case importation from country to country is sufficiently frequent, the varying seasonality of heightened transmission across latitudes could very well make regional persistence more likely than otherwise, and regional control more challenging in the absence of coordinated efforts 25 ^,^ 40 ^,^ 41. One important caveat to bear in mind, however, is that realized patterns of transmission depend not just on the potential for transmission but also on the presence of sufficient numbers of infectious and susceptible individuals in the same place at roughly the same time 26. The landscape of CHIKV transmission in the Americas is therefore likely to remain highly dynamic as its invasion progresses.

In addition to providing insight about relative patterns of transmission potential in space and time, our results also provide estimates of the magnitude of transmission potential by way of the basic reproductive number *R*_0_. In mid-latitude locations where transmission potential is expected to be greatest, our projections of yearly averages of *R*_0_ range 4-7 and projections of yearly maxima in some countries exceed 8. On the other extreme, for high- and low-latitude countries, such as Canada, the United States, Chile, Argentina, and the Falkland Islands, we projected weekly values of *R*_0_ below 1 for nearly all weeks of a typical year. Given that our analysis did not account for variation below the level of countries, there could very well be local areas within some of these countries with *R*_0_ > 1 for much of the year. On the whole, these estimates appear somewhat high compared to previous estimates, although not completely out of the realm of possibility. Using different methodology, estimates of *R*_0_ early in the invasion of CHIKIV in the Americas ranged 2-4 based on data from Guadeloupe, Martinique, and Saint Martin 13. Estimates based on data from outside the Americas 42 ^,^ 43 or on temperature-dependent parameterization of an a priori formula 12 were in the range of 4-7. It is also relevant to note that estimates of *R*_0_ for dengue, which is ecologically very similar to Chikungunya, typically range 2-6 44, with substantial seasonal variation having been noted 45. One reason that our estimates may skew high is due to our relatively low estimate of α = 0.74 (cf. α ≈ 0.9 for dengue in Thailand 27) and an inherent tradeoff between mixing and transmission 29. It is also possible that our estimates were affected by systematic errors in the data, such as reporting a backlog of cases in a single week, or failing to detect low numbers of cases early in the invasion of a given country.

As encouraging as it was that we were able to infer biologically plausible relationships between transmission and putative drivers based directly on weekly case reports, there are a number of limitations of the data and model that we used. Foremost among these limitations is the coarse spatial resolution of both. Whether it be at the level of a state or municipality, spatial disaggregation of the data would be extremely valuable for efforts to model and forecast CHIKV transmission 29 ^,^ 46, because the data could then be linked with much more relevant information about putative drivers 47. Even so, developments in modeling methodology to account for subnational heterogeneity in generating national-level patterns could possibly help in this regard. In addition to spatial and temporal resolution and other issues of data quality, coordinated efforts to make case data publicly available, and to do so in usable formats (e.g., csv rather than pdf files), would accelerate the development and application of innovative modeling and forecasting frameworks 32. The same is true for data about covariates, such as various attributes of temperature, precipitation, humidity, land cover, human population density, and others that currently require assembling from a wide range of disparate sources as well as substantial processing to make them coherent and comparable. Lastly, integrating data and models into readily usable, interactive tools that enable real-time forecasting and decision making should represent a penultimate goal of these activities, as exemplified by efforts by the United States Centers for Disease Control (www.cdc.gov/chikungunya/modeling/).

**Map indicating the minimum weekly value of R0 over a typical year for each of 53 countries.**
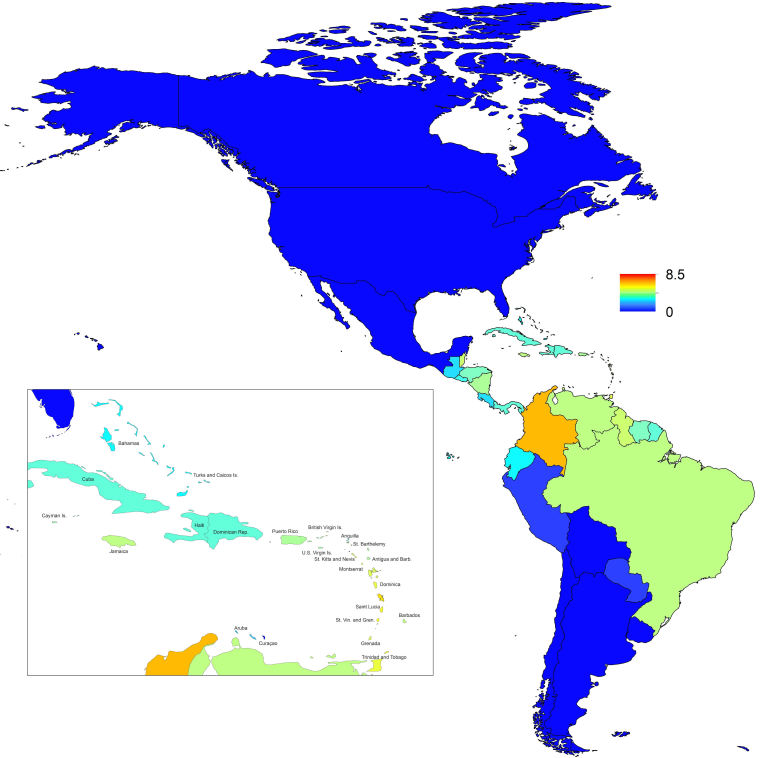

**Map indicating the mean weekly value of R0 over a typical year for each of 53 countries.**
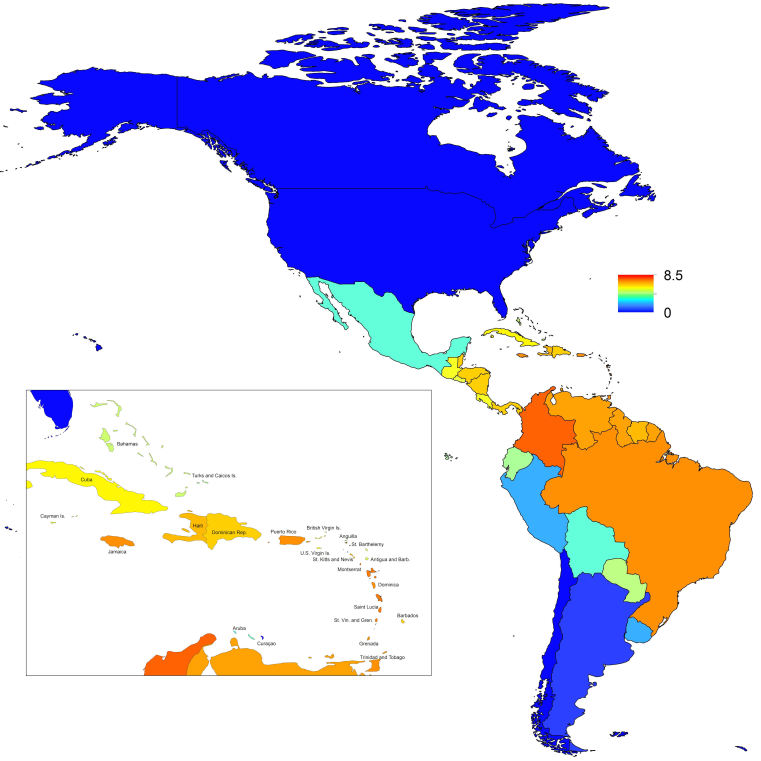

**Map indicating the maximum weekly value of R0 over a typical year for each of 53 countries.**